# Altered synaptobrevin-II trafficking in neurons expressing a synaptophysin mutation associated with a severe neurodevelopmental disorder

**DOI:** 10.1016/j.nbd.2017.08.021

**Published:** 2017-12

**Authors:** Callista B. Harper, Grazia M.S. Mancini, Marjon van Slegtenhorst, Michael A. Cousin

**Affiliations:** a Centre for Integrative Physiology, Hugh Robson Building, University of Edinburgh, Edinburgh EH8 9XD, United Kingdom; b Simonds Initiative for the Developing Brain, Hugh Robson Building, University of Edinburgh, Edinburgh EH8 9XD, United Kingdom; c Department of Clinical Genetics, Erasmus University Medical Center, 3015CN Rotterdam, The Netherlands

**Keywords:** SV, synaptic vesicle, sybII, synaptobrevin II, CV, coefficient of variation, vGLUT1, vesicular glutamate transporter 1, SNARE, soluble *N*-ethylmaleimide sensitive factor attachment protein receptor, mOr2, mOrange2, Synaptic vesicle, Endocytosis, Neuron, Nerve terminal, Synaptophysin, Synaptobrevin

## Abstract

Following exocytosis, synaptic vesicles (SVs) have to be reformed with the correct complement of proteins in the correct stoichiometry to ensure continued neurotransmission. Synaptophysin is a highly abundant, integral SV protein necessary for the efficient retrieval of the SV SNARE protein, synaptobrevin II (sybII). However the molecular mechanism underpinning synaptophysin-dependent sybII retrieval is still unclear. We recently identified a male patient with severe intellectual disability, hypotonia, epilepsy and callosal agenesis who has a point mutation in the juxtamembrane region of the fourth transmembrane domain of synaptophysin (T198I). This mutation had no effect on the activity-dependent retrieval of synaptophysin that was tagged with the genetically-encoded pH-sensitive reporter (pHluorin) in synaptophysin knockout hippocampal cultures. This suggested the mutant has no global effect on SV endocytosis, which was confirmed when retrieval of a different SV cargo (the glutamate transporter vGLUT1) was examined. However neurons expressing this T198I mutant did display impaired activity-dependent sybII retrieval, similar to that observed in synaptophysin knockout neurons. Interestingly this impairment did not result in an increased stranding of sybII at the plasma membrane. Screening of known human synaptophysin mutations revealed a similar presynaptic phenotype between T198I and a mutation found in X-linked intellectual disability. Thus this novel human synaptophysin mutation has revealed that aberrant retrieval and increased plasma membrane localisation of SV cargo can be decoupled in human disease.

## Highlights

• A novel synaptophysin variant has been identified in human disease.
• The mutation selectively perturbs activity-dependent synaptobrevin II retrieval.
• It reveals plasma membrane expression can be decoupled from vesicle cargo retrieval.

## Graphical abstract

**Figure:**
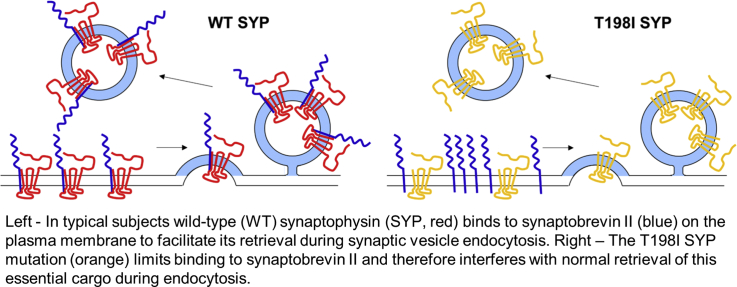

## 1 Introduction

Maintaining neurotransmission is essential for normal brain communication. Furthermore a large body of evidence is emerging indicating that altered synaptic transmission can result in a series of neurodegenerative and neurodevelopmental disorders (Milnerwood and Raymond, 2010, Sheng et al., 2012, Zoghbi and Bear, 2012). In particular synaptic dysfunction has been increasingly associated the genesis and presentation of neurodevelopmental disorders (Wijetunge et al., 2013, Zoghbi and Bear, 2012). The majority of these studies have focused on postsynaptic dysfunction, however several recent studies have highlighted presynaptic dysfunction as a potentially causal mechanism (Giovedi et al., 2014, Valente et al., 2016, Waites and Garner, 2011).

Presynaptic function is reliant on the accurate and efficient fusion (exocytosis) and reformation of synaptic vesicles (SVs) by endocytosis (Jahn and Fasshauer, 2012, Saheki and De Camilli, 2012, Sudhof, 2012). Recent studies have highlighted mutations in a series of key presynaptic genes that result in altered neurotransmitter release due to either perturbations in the number of SVs available for fusion (Corradi et al., 2014, Deng et al., 2011, Fassio et al., 2011, Myrick et al., 2015), coupling of SV fusion to calcium influx (Baker et al., 2015, Ferron et al., 2014, Valente et al., 2016, Whittaker et al., 2015), or the efficiency of the SV fusion event itself (Deciphering Developmental Disorders, 2015, Saitsu et al., 2008). In addition mutations in a number of SV endocytosis genes have been linked to neurodevelopmental disorders (Deciphering Developmental Disorders, 2015, Dhindsa et al., 2015, Euro et al., 2014, Serajee and Huq, 2015) suggesting accurate SV formation is equally important for normal presynaptic function.

Included in the endocytosis genes linked to neurodevelopmental disorders is *SYP*, which encodes the abundant SV protein synaptophysin. Synaptophysin is an integral SV protein with four transmembrane domains and cytoplasmic N- and C-termini (Sudhof et al., 1987). It is the second most abundant protein on SVs, with the most abundant being the essential fusion molecule synaptobrevin II (sybII) (Takamori et al., 2006, Wilhelm et al., 2014). Synaptophysin interacts with sybII in both mammalian brain lysates and isolated SVs. This association is postulated to occur via their respective transmembrane domains (Becher et al., 1999, Edelmann et al., 1995, Felkl and Leube, 2008, Reisinger et al., 2004, Yelamanchili et al., 2005). Neurons from synaptophysin knockout mice display a specific defect in sybII retrieval during SV endocytosis and an increased proportion of sybII mislocalised to the plasma membrane, suggesting this interaction is essential for accurate sybII trafficking (Gordon and Cousin, 2013, Gordon et al., 2011). Recent structural studies suggest that synaptophysin and sybII interact in a 1:2 ratio (Adams et al., 2015) indicating that this interaction may control the stoichiometry of their retrieval during SV endocytosis (Gordon et al., 2016).

A series of mutations in the *SYP* gene have been identified in patients with X-linked intellectual disability (Tarpey et al., 2009). These mutations were all unable to support normal sybII retrieval when expressed in synaptophysin knockout neurons (Gordon and Cousin, 2013). Thus altered sybII retrieval due to synaptophysin dysfunction may be a potentially common mechanism underlying specific neurodevelopmental disorders. We report here a novel *SYP* mutation identified in a patient with a severe neurodevelopmental disorder. This mutation does not impact on SV endocytosis or the trafficking of common SV cargoes. However it is unable to support sybII retrieval in synaptophysin knockout neurons. Intriguingly it fully restores plasma membrane levels of sybII, suggesting that activity-dependent trafficking of sybII is specifically affected by this mutation.

## 2 Methods

### 2.1 Genomic analysis of whole exome sequencing data (trio analysis)

DNA extracted from blood of the patient and both parents was enriched with Agilent Sureselect Clinical Research Exome (CRE) Capture and samples were run on the Illumina HiSeq platform. As an average, 50 million reads per exome and a mapped fraction above 98% were obtained. The average coverage is approximately 50 fold. Data were demultiplexed by Illumina software bcl2fastq. Reads were mapped to the genome using BWA (bio-bwa.sourceforge.net). Variant detection was performed by Genome Analysis Toolkit (www.broadinstitute.org/gatk). Analysis of variants was performed in Cartagenia using The Variant Calling File (VCF) followed by filtering for de novo, X-linked, recessive and dominant inheritance in a panel of 528 genes confirmed to be involved in neurodevelopmental disorders (Gilissen et al., 2014). The variant found in SYP (c.593C > T, p.Thr198Ile (SYP, exon 05)) on the X chromosome, was confirmed by capillary Sanger sequencing in DNA from the patient and the mother (in heterozygosity) while was not found in the father.

### 2.2 Materials

SybII-pHluorin was provided by Prof. G. Miesenbock (Oxford University, UK). Synaptophysin-pHluorin was from Prof. Leon Lagnado (Sussex University, UK), whereas vGLUT-pHluorin was from Prof. Robert Edwards (University of California, USA). The T198I mutant was introduced into rat synaptophysin-pHluorin by mutagenesis using the primers (mutated bases underlined) CTGAGGGACCCTGTGATTTCAGGACTCAACACC (forward) and GGTGTTGAGTCCTGAAATCACAGGGTCCCTCAG (reverse). Human synaptophysin tagged at its N-terminus with mCerulean (mCer-hSyp) was generated as outlined previously (Gordon and Cousin, 2013). The T198I mutant was introduced into mCer-hSyp by mutagenesis using the primers (mutated bases underlined) TGAGAGACCCTGTGATCTCGGGACTCAACAC (forward) and GTGTTGAGTCCCGAGATCACAGGGTCTCTCA (reverse). SybII-mOrange2 was generated by amplifying the coding sequence of mouse sybII (from sybII-pHluorin) using the primers ATTGTCTCGAGATGTCGGCTACCGCTGCCACCGTCC (forward) and CGTGTTGGATCCCGAGTGCTGAAGTAAACGATGATGATG (reverse, restriction sites underlined). The sybII sequence was cloned into a mOrange2-N1 vector (Addgene, clone number 54568) using *Xho*I and *Bam*HI enzymes.

Neurobasal media, B-27 supplement, penicillin/streptomycin, Minimal Essential Medium (MEM), and Lipofectamine 2000 were obtained from Invitrogen (Paisley, UK). All other reagents were obtained from Sigma-Aldrich (Poole, UK).

### 2.3 Hippocampal neuronal cultures

Synaptophysin knockout mice were maintained as heterozygous breeding pairs on a C57/BL6J background (Gordon et al., 2011). All animal work was performed in accordance with the UK Animal (Scientific Procedures) Act 1986, under Project and Personal License authority and was approved by the Animal Welfare and Ethical Review Body at the University of Edinburgh. All animals were killed by schedule 1 procedures in accordance with UK Home Office Guidelines. Dissociated primary hippocampal enriched neuronal cultures were prepared from E16.5-18.5 embryos from either synaptophysin knockout or wild-type C57/BL6J mice of both sexes as outlined (Zhang et al., 2015). Single-cell suspension of hippocampal neurons were plated at a density of 3–5 × 10^4^ cells/coverslip on poly-d-lysine and laminin-coated 25 mm coverslips. Cells were transfected after 7–8 days in culture with Lipofectamine 2000 as described (Gordon and Cousin, 2013). Cells were imaged after 13–16 days in culture.

### 2.4 Fluorescent imaging protocols

Hippocampal cultures were mounted in a Warner Instruments (Hamden, CT, USA) imaging chamber with embedded parallel platinum wires (RC-21BRFS) and placed on the stage of Zeiss (Cambridge, UK) Axio Observer D1 inverted epifluorescence microscope. Neurons transfected with mCer vectors were visualised with a Zeiss EC Plan Neofluar 40 ×/1.30 oil immersion objective at 430 nm excitation, whereas pHluorin reporters were visualised at 500 nm excitation. The same emission collection was applied in both instances (using a 515 nm dichroic filter and long-pass emission filter, > 520 nm). Dual colour pHluorin and mOrange2 images were acquired on a dual camera system and the signal filtered using a double band pass excitation filter (470/27 + 556/25) with beam splitter (490 + 575) and emission filters 512/30 and 630/98 (Zeiss). Fluorescent images were captured at 4 s intervals using an AxioCam 506 mono digital camera (Zeiss). Cultures were stimulated with a train of 300 action potentials delivered at 10 Hz (100 mA, 1 ms pulse width) during which there was a continuous perfusion of imaging buffer (in mM: 119 NaCl, 2.5 KCl, 2 CaCl2, 2 MgCl2, 30 d-glucose, 25 HEPES, pH 7.4 supplemented with 10 μM 6-cyano-7-nitroquinoxaline-2,3-dione and 50 μM DL-2-Amino-5-phosphonopentanoic acid), and were then challenged with an alkaline imaging buffer (50 mM NH_4_Cl substituted for 50 mM NaCl) to reveal total pHluorin fluorescence.

Surface-localised sybII-pHluorin reporter was revealed by bathing neurons in imaging buffer, and then perfusing with an acidic buffer (2-(*N*-morpholino)ethanesulfonic acid substituted for HEPES, pH 5.5) to quench fluorescence from sybII-pHluorin present on the plasma membrane and retain background autofluorescence. Neurons were washed in imaging buffer, and then exposed to an alkaline imaging buffer to reveal total sybII-pHluorin fluorescence.

### 2.5 Data processing

Offline processing was performed using Fiji is just ImageJ (Fiji) software (Schindelin et al., 2012). Regions of interest of identical size were placed over nerve terminals and the fluorescence intensity was monitored over time using the Time Series Analyzer plugin. Regions of interest were then screened using a customised Java program that allows for visualisation of the fluorescent responses and removal of the data from nerve terminals that did not respond or displayed no recovery following termination of the stimuli.

All subsequent data analyses were performed using Microsoft Excel, Matlab (Cambridge, UK) and GraphPad Prism 6.0 (La Jolla, CA, USA) software. The change in activity-dependent pHluorin fluorescence was calculated as ΔF/F0 and normalised to the peak during stimulation or alkaline buffer perfusion, while the surface fraction of sybII-pHluorin expressed as a percentage of total pHluorin was calculated using the following equation: [(basal fluorescence − acidic fluorescence)/(alkaline fluorescence − acidic fluorescence)] × 100.

The distribution of sybII-pHluorin fluorescence along axons was determined by calculating the coefficient of variation (CV), which was measured while neurons were exposed to alkaline imaging buffer to reveal total pHluorin fluorescence. The standard deviation/mean fluorescence intensity (CV) was calculated from a line profile along axon segments expressing pHluorin reporters. The mean of 5 different > 100 pixel axonal segments on a single coverslip was calculated for each experiment.

Dual colour data was analysed by first calculating ΔF/F0 values and correcting for photobleaching using a single exponential function. The data was then analysed as described above for sybII-pHluorin/-mOr2. Alternatively, to examine the responses of sybII-mOr2 and syp-pHluorin in individual nerve terminals, fluorescent traces from single regions of interest were normalised to the stimulation peak prior to filtering and removal of non-responding nerve terminals. The first 10 frames of decay following stimulation were then fit to a linear function to obtain the initial slope for each fluorescent trace post-stimulation. In addition, the mean value of the final 5 frames (184–200 s) for each nerve terminal was determined to calculate the fraction remaining on the surface at the end of the experiment. The sybII-mOr2 responses were then plotted against the corresponding syp-pHluorin response from each nerve terminal and fit to a linear function to examine the relationship between the kinetics of sybII-mOr2 and syp-pHluorin retrieval.

### 2.6 Statistical analysis

All statistical analysis was performed in Graph Pad Prism 6.0. A one-way ANOVA with Tukey's post-test were used to compare more than two groups, while a repeated measures two-way ANOVA with Tukey's multiple-comparison post-test was performed to compare multiple groups over time. Unless stated otherwise, the sample size (*n*) was taken to be the number of independent experiments or individual coverslips. All data are presented as mean values ± standard error of the mean (SEM).

## 3 Results

### 3.1 Case report

A boy was born after a full term pregnancy with evident micropenis and cryptorchidism. At the age of seven months hypogonadotropic hypogonadism was diagnosed and he was referred to a child neurologist because of hypotonia, restless movements and lack of visual awareness. An MRI scan revealed complete agenesis of the corpus callosum. In the suspicion of a Kallmann-like syndrome and Prader-Willi syndrome, genetic analysis was performed, but did not confirm any of these syndromes. At the age of 1.5 years he developed generalised seizures with focal EEG abnormalities, which were well controlled by valproate therapy. His neuromotor development remained strongly delayed, at the age of 9 years he did not reach the ability to stand or walk, he developed scoliosis, divergent strabism and feedings difficulties. Whole exome sequencing revealed a maternally inherited missense change in *SYP*, leading to an amino acid substitution, p.Thr198Ile (here abbreviated as T198I).

### 3.2 Neurons expressing hSyp_T198I_ display normal SV recycling

**Fig. 1:**
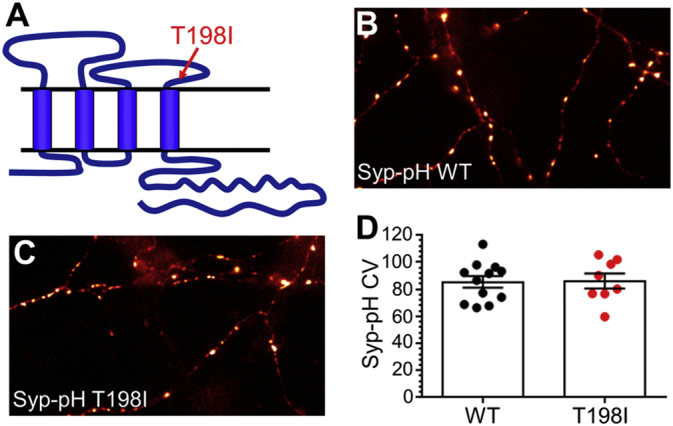
The T198I mutation does not impact on synaptophysin localisation. (A) Schematic of the structure of synaptophysin with the location of the T198I mutation indicated by an arrow. (B–D) Primary cultures of hippocampal neurons derived from synaptophysin knockout mice were transfected with either wild-type (WT) or T198I mutant synaptophysin-pHluorin (Syp-pH). Representative images of neurons transfected with either syp_WT_-pHluorin (B) or syp_T198I_-pHluorin (C) are displayed, which have been pseudo-coloured to indicate regions of high intensity in white. Scale bar equivalent to 10 μm. (D) Coefficient of variation (CV) analysis for either syp_WT_-pHluorin (Black) or syp_T198I_-pHluorin (red) is displayed ± SEM (WT *n* = 12, T198I *n* = 8; students *t*-test *p* = 0.92).

The T198I mutation resides juxtaposed to the fourth transmembrane domain in synaptophysin (Fig. 1A). Recent structural studies have identified this region as potentially important for sybII binding (Adams et al., 2015), suggesting this mutant may display dysfunctional sybII retrieval. To determine this, a series of genetically-encoded fluorescent reporters of SV cargo trafficking were expressed in primary cultures of hippocampal neurons derived from synaptophysin knockout mice. These reporters are termed pHluorins, and are comprised of an exogenously-expressed SV cargo with a pH-sensitive EGFP moiety fused to a lumenal domain (Kavalali and Jorgensen, 2014). The fluorescence of these reporters is quenched in the acidic environment of the SV, whereas it is unquenched in neutral environments such as the plasma membrane. This allows both static measurement of the distribution of SV cargo at different synaptic compartments and an accurate method to track their activity-dependent movement, since fluorescence is unquenched on SV fusion and quenched on retrieval and SV acidification. Since SV endocytosis is rate limiting when compared to SV acidification, fluorescence quenching can be used as an estimate of the speed of SV cargo retrieval (Atluri and Ryan, 2006, Egashira et al., 2015, Granseth et al., 2006).

**Fig. 2:**
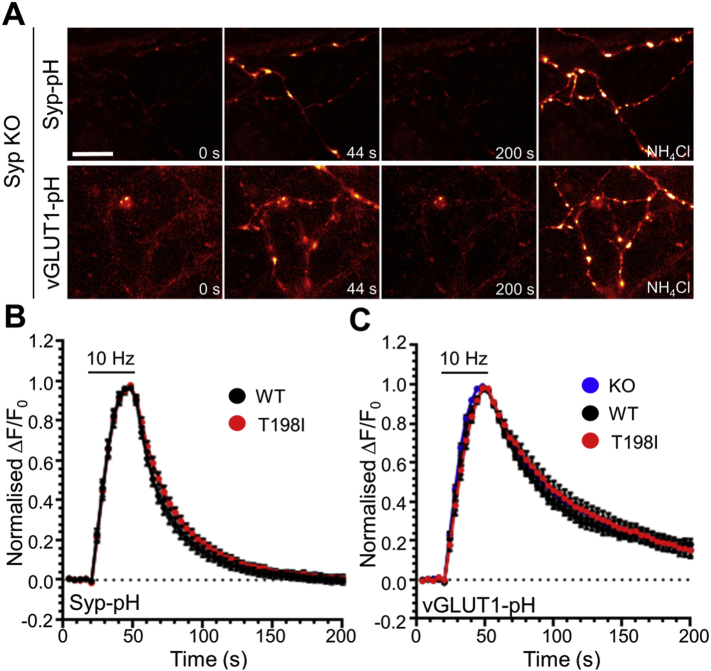
The T198I mutation does not impact on synaptic vesicle endocytosis. Primary cultures of hippocampal neurons derived from synaptophysin knockout mice were transfected with either (A, B) wild-type (WT) or T198I mutant synaptophysin-pHluorin (Syp-pH) or (A, C) vGLUT1-pHluorin and either wild-type mCerulean-tagged human synaptophysin (WT), the T198I mutant (T198I) or empty mCer vector (KO). Neurons were then challenged with a train of 300 action potentials delivered at 10 Hz before perfusing buffer containing NH_4_Cl. (A) Pseudo-coloured images from a representative time-lapse are shown prior to the stimulus (t = 0 s), during the stimulus (t = 44 s), following endocytosis (t = 200 s) and during de-quenching of the vesicular pool of pHluorin by NH_4_Cl. Images have been pseudo-coloured to indicate regions of high intensity in white. Scale bar equivalent to 10 μm. (B) Syp_WT_-pHluorin (black) or syp_T198I_-pHluorin (red) traces display the average fluorescent syp-pHluorin response normalised to the peak of stimulation (ΔF/F_0_ ± SEM, WT *n* = 12, T198I *n* = 8). (C) The average fluorescent vGLUT1-pHluorin response normalised to the peak of stimulation (ΔF/F_0_ ± SEM) is displayed for neurons expressing mCer-hSyp_WT_ (black), mCer-hSyp_T198I_ (red) or mCer (KO, blue). WT *n* = 9, T198I *n* = 8, KO *n* = 15. In both cases the bar indicates the period of stimulation. A two-way ANOVA was performed and no significant difference was observed between the traces.

We first examined whether the T198I mutation impacted on the targeting of synaptophysin to nerve terminals using synaptophysin-pHluorin (syp-pHluorin). To quantify this parameter, the coefficient of variation (CV) was analysed. CV quantifies protein localisation, with a lower CV indicating a homogeneous expression along the axon, and a higher CV signifying a more punctate distribution (Lyles et al., 2006), such as at nerve terminals. When this analysis was performed, the CV of syp_T198I_-pHluorin showed no significant difference to that of syp_WT_-pHluorin (Fig. 1B–D) indicating the T198I mutation did not interfere with the ability of synaptophysin to be efficiently targeted to nerve terminals. We next examined whether this mutation altered the activity-dependent trafficking of synaptophysin. Synaptophysin knockout cultures expressing either syp_WT_-pHluorin or syp_T198I_-pHluorin were challenged with a train of 300 action potentials delivered at 10 Hz. The syp_WT_-pHluorin reporter displayed a characteristic response, with an increase in fluorescence during stimulation corresponding to SV fusion (Fig. 2A, B). Following termination of the stimulus the fluorescent response of syp_WT_-pHluorin rapidly decreased, representative of the kinetics of synaptophysin retrieval. When the activity-dependent syp_T198I_-pHluorin response was assessed, there was no significant difference when compared to syp_WT_-pHluorin (Fig. 2B). Thus the T198I mutation does not impact on the nerve terminal targeting or activity-dependent trafficking of synaptophysin.

The absence of effect of the T198I mutation on synaptophysin trafficking also suggests that SV endocytosis is unaffected by expression of this variant, since if this were the case the post-stimulation fluorescent decay of the syp_T198I_-pHluorin response would have been modified. To confirm this, we examined the trafficking of a different SV cargo, the vesicular glutamate transporter, vGLUT1 (Voglmaier et al., 2006). Synaptophysin knockout neurons were co-transfected with vGLUT1-pHluorin and either mCer-hSyp_WT_, mCer-hSyp_T198I_ or mCer empty vector and challenged with a train of 300 action potentials as before. The absence of synaptophysin had no significant effect on the retrieval of vGLUT1-pHluorin, as evidenced by the similarity of fluorescent responses in neurons expressing either mCer-hSyp_WT_ or the empty mCer vector (Fig. 2A, C). Expression of mCer-hSyp_T198I_ did not affect the vGLUT1-pHluorin response when compared to neurons expressing either mCer-hSyp_WT_ or the empty mCer vector (Fig. 2C). Therefore the T198I mutation does not perturb SV endocytosis.

### 3.3 Neurons expressing hSyp_T198I_ display a selective deficit in activity-dependent sybII traffic

**Fig. 3:**
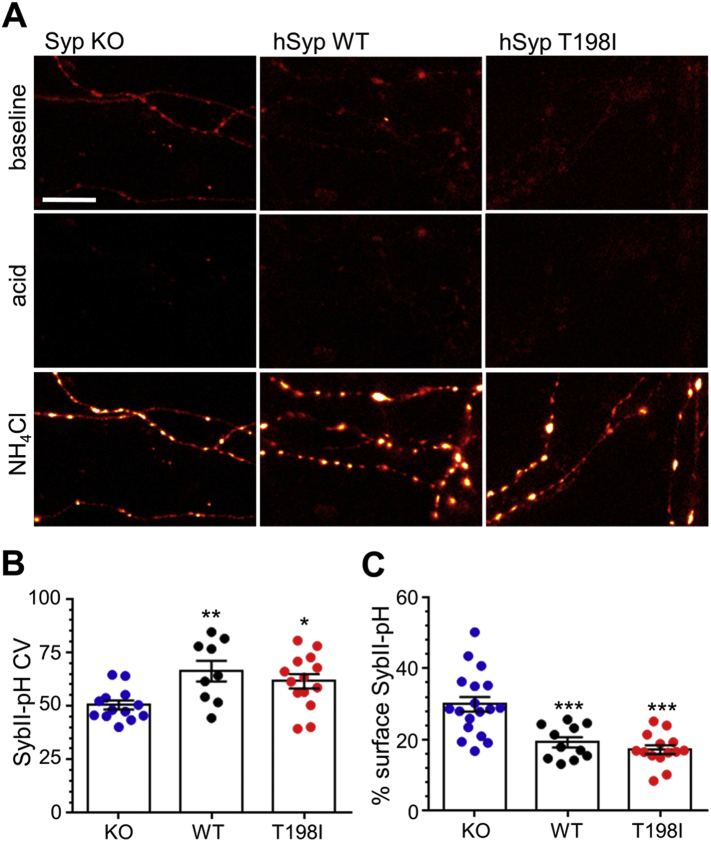
The T198I mutation has no effect on sybII localisation or surface fraction. Primary cultures of synaptophysin knockout hippocampal neurons were transfected with sybII-pHluorin and either wild-type mCerulean-tagged human synaptophysin (WT), the T198I mutant (T198I) or empty mCer vector (KO). (A) Representative images of neurons transfected with sybII-pHluorin are displayed. The images have been pseudo-coloured to indicate regions of high intensity in white. Scale bar equivalent to 10 μm. (B) Coefficient of variation (CV) analysis for sybII-pHluorin is displayed for neurons co-expressing either WT (black), T198I (red) or mCer (KO, blue) ± SEM (WT *n* = 11, T198I *n* = 14, KO *n* = 18). ** = *p* < 0.01, * = *p* < 0.05, ** = *p* < 0.01, one-way ANOVA against WT. (C) Surface expression of sybII-pHluorin is displayed as a percentage of total sybII-pHluorin for neurons co-expressing WT (black), T198I (red) and mCer (KO, blue) ± SEM (WT *n* = 11, T198I *n* = 14, KO *n* = 18). *** = *p* < 0.001, one-way ANOVA against WT.

The absence of synaptophysin causes a mislocalisation of its interaction partner sybII from nerve terminals with both endogenous and exogenous sybII displaying a diffuse axonal distribution (Gordon and Cousin, 2013, Gordon et al., 2011). Therefore we next determined whether replacing endogenous synaptophysin with the T198I mutant impacted on the targeting of sybII-pHluorin to synaptophysin knockout nerve terminals. As observed previously, there was a significant loss of sybII-pHluorin fluorescence from nerve terminals expressing the empty mCer vector when quantified by CV (Fig. 3A, (Gordon and Cousin, 2013, Gordon et al., 2011)). In contrast neurons expressing mCer-hSyp_WT_ displayed a more punctate distribution and a significantly higher CV value (Fig. 3A, B). When neurons expressing mCer-hSyp_T198I_ were examined, sybII-pHluorin was targeted in an equivalent manner to those expressing mCer-hSyp_WT_ (Fig. 3A, B). Therefore the T198I mutation in synaptophysin does not impact on the presynaptic targeting of sybII.

The principal presynaptic deficit in the absence of synaptophysin is aberrant retrieval of sybII from the plasma membrane during SV endocytosis (Gordon and Cousin, 2013, Gordon et al., 2011). This is usually reflected by an increased presence of sybII-pHluorin at the presynaptic plasma membrane and a retardation of its activity-dependent retrieval. To determine whether the T198I mutation impacts on sybII retrieval, we first examined the effect of replacing endogenous synaptophysin with mCer-hSyp_T198I_ on the plasma membrane expression of sybII-pHluorin. This was determined by application of an impermeant acidic buffer to quench sybII-pHluorin exposed to the extracellular space. This plasma membrane fraction was expressed as a proportion of total sybII-pHluorin, which was revealed by dequenching all fluorescence with an ammonium chloride solution. The absence of synaptophysin resulted in the stranding of sybII-pHluorin at the cell surface when compared to rescue with mCer-hSyp_WT_ (Fig. 3A, C), as observed previously (Gordon et al., 2011). Expression of mCer-hSyp_T198I_ fully rescued sybII-pHluorin surface expression in synaptophysin knockout neurons (Fig. 3A, C), indicating that there is no localisation deficit in the presence of this mutant in resting neurons.

**Fig. 4:**
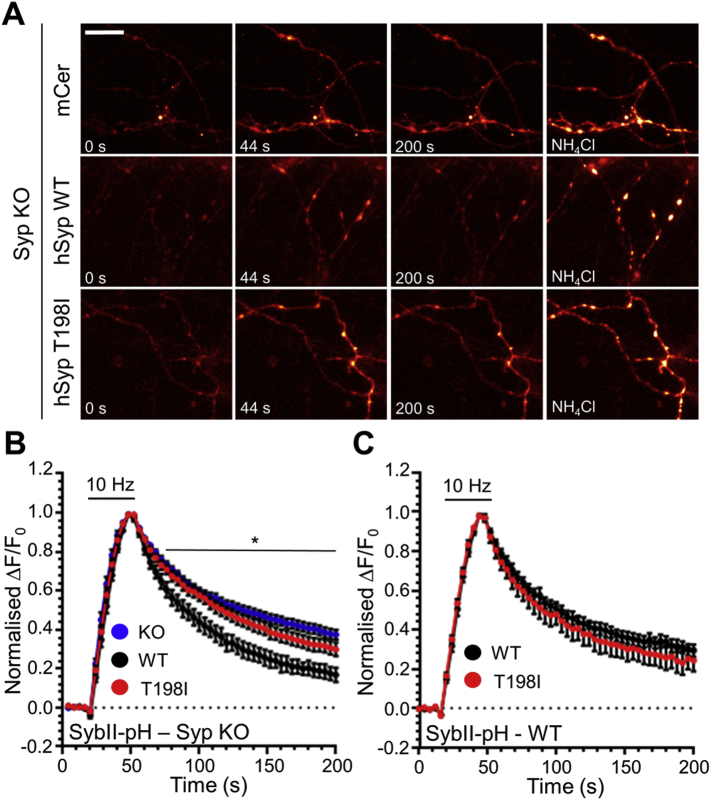
The T198I mutation selectively impacts on activity-dependent sybII retrieval during SV endocytosis. (A, B) Primary cultures of synaptophysin knockout hippocampal neurons were transfected with sybII-pHluorin and either wild-type mCerulean-tagged human synaptophysin (WT), the T198I mutant or empty mCer vector (KO) and challenged with a train of 300 action potentials (10 Hz) before being allowed to recover and exposed to a NH_4_Cl containing buffer. (A) Pseudo-coloured images from a representative time-lapse are shown prior to the stimulus (t = 0 s), during the stimulus (t = 44 s), following endocytosis (t = 200 s) and during perfusion of NH_4_Cl. Scale bar equivalent to 10 μm. (B) Traces (WT, black; T198I, red; KO, blue) display the average fluorescent sybII-pHluorin response normalised to the peak of stimulation (ΔF/F_0_ ± SEM). Stimulation is indicated by the bar. WT *n* = 9, T198I *n* = 14, mCer *n* = 13, * = *p* < 0.05 for both T198I and KO compared to WT, two-way ANOVA. (C) Wild-type hippocampal neurons transfected with sybII-pHluorin and either WT (black) or T198I (red) mCerulean-tagged human synaptophysin were challenged with a train of 300 action potentials (10 Hz) as indicated by the bar. Traces display the average fluorescent sybII-pHluorin response normalised to the peak of stimulation (ΔF/F_0_ ± SEM). WT *n* = 11, T198I *n* = 10, ns, two-way ANOVA.

**Fig. 5:**
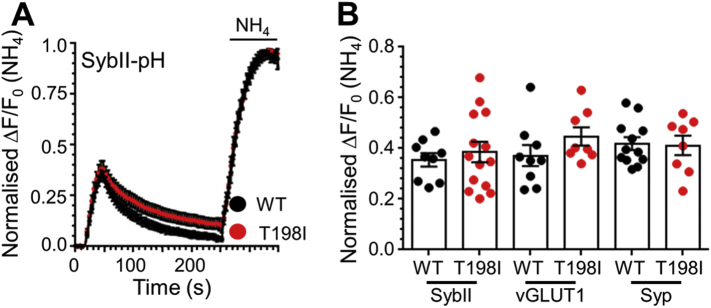
The T198I mutation has no effect on SV exocytosis. Primary cultures of synaptophysin knockout hippocampal neurons were transfected with either WT (black) or T198I (red) syp-pHluorin (B) or co-transfected with sybII-pHluorin (A, B) or vGLUT1-pH (B) and wild-type mCerulean-tagged human synaptophysin (WT, black) or the T198I mutant (T198I, red). Neurons were challenged with a train of 300 action potentials (10 Hz) and after 3 min a pulse of a buffer containing 50 mM NH_4_Cl to unquench all pHluorin fluorescence was applied as indicated by bars. (A) Traces display the average fluorescent sybII-pHluorin response normalised to the total pool (ΔF/F_0_ (NH_4_) ± SEM). (B) The average peak height of sybII-pHluorin (sybII), vGLUT1-pHluorin (vGLUT1) or syp-pHluorin (syp) normalised to NH_4_Cl (ΔF/F_0_ (NH_4_) ± SEM) is displayed (sybII-pH; WT *n* = 9, T198I *n* = 14, vGLUT-pH; WT *n* = 9, T198I *n* = 8, syp-pH WT *n* = 12, T198I *n* = 8; all ns students *t*-test).

To determine whether the activity-dependent trafficking of sybII-pHluorin was affected by this mutation, synaptophysin knockout neurons expressing either mCer-hSyp_WT_, mCer-hSyp_T198I_ or mCer empty vector were challenged with a train of 300 action potentials. The absence of synaptophysin resulted in a slowing of the retrieval of sybII-pHluorin when compared to rescue with mCer-hSyp_WT_ (Fig. 4A, B), as observed previously (Gordon and Cousin, 2013, Gordon et al., 2011). Intriguingly mCer-hSyp_T198I_ did not rescue sybII-pHluorin retrieval kinetics, instead sybII-pHluorin retrieval appeared very similar to that observed in the absence of synaptophysin (Fig. 4A, B). This was not due to a change in the number of SVs fusing by exocytosis, since the proportion of sybII-pHluorin undergoing exocytosis was unchanged when normalised to the total sybII-pHluorin pool (Fig. 5A, B). This was also the case for syp-pHluorin and vGLUT1-pHluorin (Fig. 5B). Thus there is a specific, activity-dependent deficit in sybII retrieval when endogenous synaptophysin is replaced by the T198I synaptophysin mutant.

To determine whether this activity-dependent defect in sybII traffic observed with the T198I mutant was due to loss of synaptophysin function, we expressed mCer-hSyp_T198I_ in wild-type neurons to determine whether it had any dominant negative effect. There was no difference in activity-dependent sybII-pHluorin trafficking in cells expressing mCer-hSyp_T198I_ when compared to those expressing mCer-hSyp_WT_ (Fig. 4C), therefore the T198I mutation is a loss of function mutation.

**Fig. 6:**
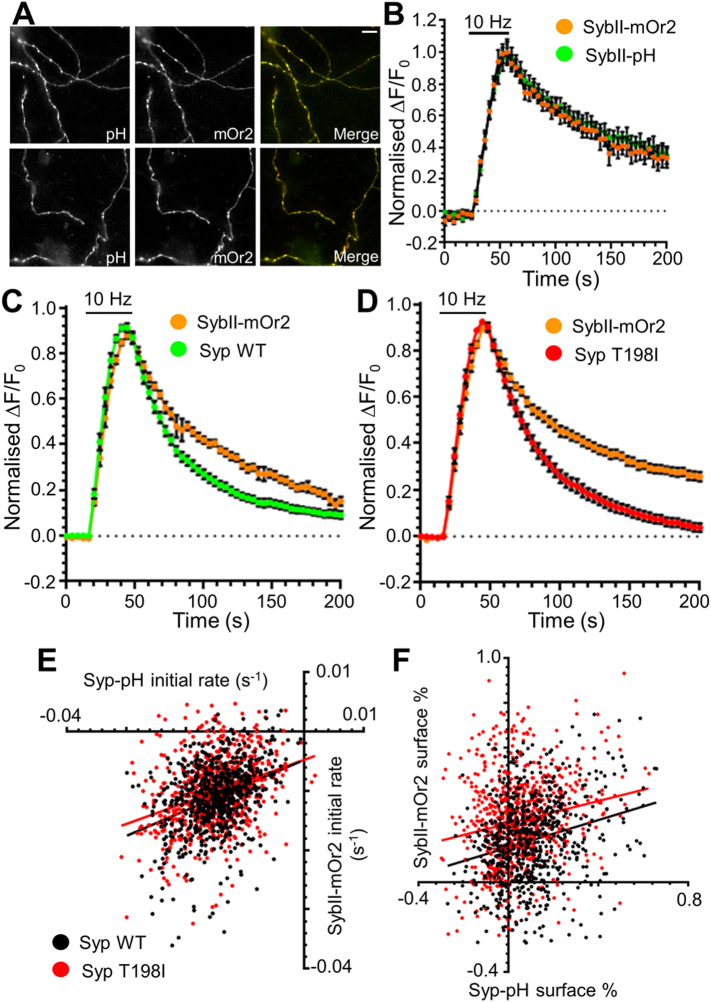
Dual colour imaging of synaptophysin and sybII trafficking during SV endocytosis. (A, B) Primary cultures of wild-type hippocampal neurons were transfected with sybII-mOrange2 (sybII-mOr2) and sybII-pHluorin (sybII-pH). (A) Images display the fluorescence of either sybII-pHluorin (pH, green), sybII-mOr2 (mOr2, red) or a merged image. Scale bar equivalent to 10 μm. (B) Neurons co-expressing sybII-pHluorin and sybII-mOr2 were challenged with a train of 300 action potentials (10 Hz). SybII-mOr2 (orange) and sybII-pHluorin (green) traces display the average fluorescent response normalised to the peak of stimulation (ΔF/F_0_ ± SEM, both *n* = 8). Bar indicates the period of stimulation. (C, D) Synaptophysin knockout hippocampal neurons were transfected with sybII-mOr2 and either wild-type (WT) or T198I mutant synaptophysin-pHluorin (Syp-pH) and challenged with a train of 300 action potentials (10 Hz). SybII-mOr2 (orange), syp_WT_–pHluorin (green) and syp_T198I_–pHluorin (red) traces display the average fluorescent response of each nerve terminal normalised to the peak of stimulation (ΔF/F_0_ ± SEM). Bar indicates period of stimulation (WT *n* = 13, T198I *n* = 15). * = *p* < 0.05 for sybII-mOr2 response when comparing WT and T198I expressing neurons, two-way ANOVA. (E) The rate of decay of sybII-mOr2 fluorescence during the first 10 frames following stimulation was plotted against the corresponding syp–pHluorin values and fit to a linear function (WT, black r_2_ = 0.134; *n* = 697, T198I, red r_2_ = 0.099; *n* = 700). No significant difference in either slope (0.4447 (WT) vs. 0.3479 (T198I)) or Y-intercept (− 0.0045 (WT) vs. − 0.0050 (T198I)) was observed between sybII values in relation to syp_WT_ and syp_T198I_ (F) The fraction of sybII-mOr2 remaining on the plasma membrane 150 s following stimulation was plotted against the corresponding syp-pHluorin values and fit to a linear function (WT, black r_2_ = 0.062; *n* = 697, T198I, red r_2_ = 0.036; *n* = 700). While no difference was observed in the slope (0.304 (WT) vs. 0.252 (T198I)), the Y-intercept was significantly higher (0.155 (WT) vs. 0.261 (T198I)) (*p* < 0.0001) for sybII-mOr2 when expressed with syp_T198I_-pHluorin, indicating increased surface stranding of sybII-mOr2.

To confirm that the T198I mutation specifically impacts sybII retrieval without altering synaptophysin trafficking, we next examined the trafficking of synaptophysin and sybII using dual colour imaging. This approach monitors the activity-dependent retrieval of both syp-pHluorin and sybII fused to the pH-sensitive dsRed variant mOrange2 (mOr2) (Shaner et al., 2008), permitting tracking of the retrieval of both proteins within the same nerve terminals. To first confirm that dual colour imaging accurately reflected cargo trafficking observed using pHluorins, wild-type neurons were transfected with both sybII-pHluorin and sybII-mOr2 (Fig. 6A). The fluorescent response evoked by a train of 300 action potentials was identical between the two fluorescent proteins, indicating that the mOr2 response accurately reports sybII traffic (Fig. 6B).

To examine the trafficking of sybII-mOr2 and syp-pHluorin within the same nerve terminals, synaptophysin knockout neurons were transfected with sybII-mOr2 and either syp_WT_-pHluorin or syp_T198I_-pHluorin. Following normalisation of the individual nerve terminals to the stimulation peak, the average responses of nerve terminals expressing sybII-mOr2 and either syp_WT_-pHluorin or syp_T198I_-pHluorin were examined, with the results highly similar to those using pHluorins (Fig. 6C, D). Specifically, there was no significant difference between the syp_WT_-pHluorin and syp_T198I_-pHluorin responses, whereas there was a significant difference between the sybII-mOr2 responses in neurons expressing syp_WT_-pHluorin and those expressing syp_T198I_-pHluorin (two-way ANOVA, *p* < 0.01).

The fluorescent responses of syp_WT_-pHluorin and sybII-mOr2 from the same cells showed that although each of the proteins retrieved at a similar rate, the kinetics appeared to be distinct (Fig. 6C, D). We therefore determined whether the retrieval kinetics of synaptophysin and sybII showed a correlation at the level of individual nerve terminals. We first examined the rate of cargo retrieval in each nerve terminal by calculating the initial decay in fluorescence during the first 40 s following stimulation using a linear function. There was a good correlation between the initial retrieval kinetics of sybII-mOr2 when compared to syp_WT_-pHluorin (Fig. 6E). No change in this relationship was detected when compared to that of sybII-mOr2 and syp_T198I_-pHluorin. This indicates that within individual nerve terminals the rate of sybII retrieval is unaltered by the T198I mutation.

We next determined whether the capacity of individual nerve terminals to retrieve sybII was impacted by the synaptophysin T198I mutation. We examined whether the fraction of sybII and synaptophysin stranded on the plasma membrane after action potential stimulation was correlated across individual nerve terminals. This analysis demonstrated that nerve terminals that efficiently cleared syp_WT_-pHluorin from the plasma membrane also did so for sybII-mOr2. This relationship was again unchanged when syp_T198I_-pH was expressed, however increased sybII-mOr2 stranding was observed as evidenced by a significant increase in the Y-intercept value (Fig. 6F). This result indicates that nerve terminals expressing the synaptophysin T198I mutant display a decreased capacity for sybII retrieval, while retaining a close correlation in the proportional extent of retrieval.

### 3.4 Neurons expressing hSyp mutants impact sybII surface stranding to differing extents

**Fig. 7:**
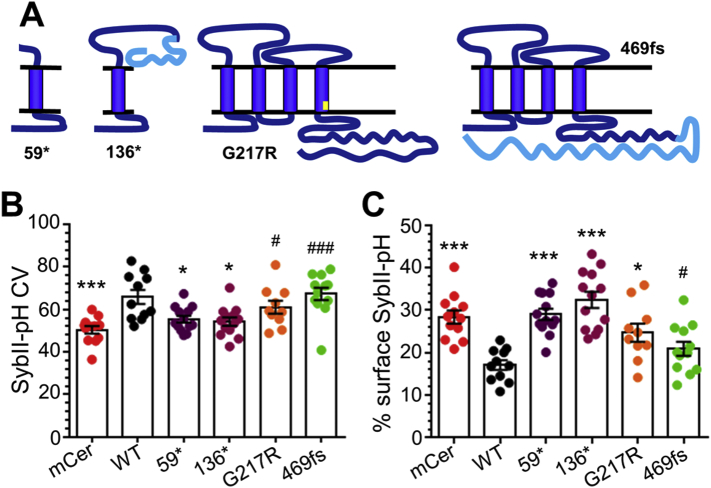
Effect of multiple XLID synaptophysin mutations on sybII targeting and surface fraction. (A, B) Primary cultures of synaptophysin knockout hippocampal neurons were transfected with sybII-pHluorin and either wild-type mCerulean-tagged human synaptophysin (WT, black), truncation mutants (59*, purple and 136*, maroon) a point mutant (G217R, orange), a frame shift mutant (469 fs, green) or empty mCer vector (mCer, red). (A) Coefficient of variation (CV) analysis for sybII-pHluorin is displayed ± SEM (mCer *n* = 12, WT *n* = 11, 59* *n* = 13, 136* *n* = 13, G217R *n* = 10, 469 fs *n* = 12). *** = *p* < 0.001, * = *p* < 0.05 one-way ANOVA compared to WT, ^###^ = *p* < 0.001, ^#^ = *p* < 0.05 one-way ANOVA compared to mCer. (B) Surface expression of sybII-pHluorin is displayed as a percentage of total ± SEM (mCer *n* = 12, WT *n* = 11, 59* *n* = 13, 136* *n* = 13, G217R *n* = 10, 469 fs *n* = 12, KO *n* = 12). *** = *p* < 0.001, * = *p* < 0.05 one-way ANOVA compared to WT, ^#^ = *p* < 0.05 one-way ANOVA compared to mCer.

The specific effect of the T198I synaptophysin mutant on activity-dependent sybII trafficking but not cell surface expression was unusual, since the latter parameter is usually the result of a perturbation in cargo retrieval. To determine whether this was a common consequence of synaptophysin dysfunction, we determined the effect of a series of synaptophysin mutations identified in patients with X-linked intellectual disability (Tarpey et al., 2009) on sybII surface expression. Two of these mutants result in the truncation of synaptophysin (59* and 136*), and replacement of the endogenous protein with these mutants could not rescue activity-dependent sybII-pHluorin retrieval (Gordon and Cousin, 2013). Replacement with the other mutants (a point mutation in the 4th transmembrane domain (G217R) and frame shift in the C-terminus, 469 fs) supported a partial recovery of sybII-pHluorin retrieval (Gordon and Cousin, 2013). We first confirmed the effect of these mutants on sybII-pHluorin targeting. We found that the truncation mutants mCer-hSyp_59⁎_ and mCer-hSyp_136⁎_ did not rescue sybII-targeting, mCer-hSyp_G217R_ displayed an intermediate rescue and mCer-hSyp_469fs_ fully rescued, in agreement with previous results (Fig. 7B) (Gordon and Cousin, 2013). When sybII-pHluorin plasma membrane stranding was evaluated, a complex picture emerged. The truncation mutants mCer-hSyp_59⁎_ and mCer-hSyp_136⁎_ did not rescue surface stranding over that observed in the absence of synaptophysin. Contrary to its ability to target sybII to nerve terminals, mCer-hSyp_G217R_ was also unable to rescue surface stranding of sybII. Conversely, mCer-hSyp_469fs_ fully rescued sybII-pHluorin surface expression to wild-type levels. Thus defects in activity-dependent sybII trafficking do not necessarily translate into plasma membrane stranding or mislocalisation of sybII along the axon.

## 4 Discussion

A novel mutation in the *SYP* gene was identified in a male patient with severe ID, hypotonia, epilepsy, hypogonadotropic hypogonadism and callosal agenesis. The combination of problems in our patient differs from the previously reported patients, because hypogonadism, severe hypotonia and callosal agenesis have not been previously described and define a recognisable, syndromic type of X-linked intellectual disability (Tarpey et al., 2009). When this mutant was expressed in a knockout system, it did not impact on synaptophysin trafficking or SV endocytosis. It did however have a selective effect on the activity-dependent retrieval of its interaction partner sybII, but not on sybII targeting to nerve terminals or plasma membrane levels. This mutation has revealed that synaptophysin-dependent control of activity-dependent sybII traffic can be decoupled from changes in sybII localisation and suggests that synaptophysin control of sybII trafficking may involve multiple mechanisms.

In addition to a selective effect on sybII trafficking, synaptophysin knockout neurons display a minor slowing in the kinetics of SV endocytosis (Kwon and Chapman, 2011, Rajappa et al., 2016). In previous studies we have also observed this phenomenon, however it did not impact on the number of SVs retrieved during an action potential train (Gordon et al., 2011). In this study we also observed a slight slowing in SV endocytosis, measured using vGLUT1-pH, both in the absence of synaptophysin and in the presence of the T198I mutant, however in both cases it did not reach significance (Time constant (τ) WT 41.4 ± 4.3 s *n* = 9; mCer 52.7 ± 5.3 s *n* = 15; T198I 63.4 ± 13.3 s *n* = 8; not significant one-way ANOVA). A recent study has suggested that this slowing defect is activity-dependent, with the absence of synaptophysin having little impact with mild stimuli (Rajappa et al., 2016). This slowing is not a secondary consequence of perturbed sybII retrieval, since normal SV endocytosis kinetics can be observed during dysfunctional sybII retrieval (Gordon and Cousin, 2013). Therefore the physiological relevance of this kinetic effect is still unclear.

Mistargeting to the plasma membrane is often a consequence of retarded SV cargo retrieval. However the human synaptophysin mutations examined in this work reveal that these events can be uncoupled. Both the T198I and 469 fs synaptophysin mutants were able to fully rescue surface stranding and localisation of sybII while still displaying an activity-dependent defect in retrieval. The most parsimonious reason for these discrepancies in trafficking and localisation is that sybII is retrieved in the presence of the mutant albeit with slower kinetics. Therefore surface stranding may only be apparent transiently following prolonged stimulation.

The lack of correlation between retarded retrieval and plasma membrane localisation of SV proteins has also been observed for a different SV cargo, synaptotagmin-1. Synaptotagmin-1 displayed accelerated activity-dependent retrieval in parallel with an increased expression at the plasma membrane when its monomeric adaptor stonin-2 or SV interaction partner SV2A were depleted from neurons (Kaempf et al., 2015, Kononenko et al., 2013, Zhang et al., 2015). Mutations such as the T198I mutation may therefore be highly informative in assembling a comprehensive picture of the relationship between activity-dependent SV cargo retrieval and cell surface fraction.

The correlation between synaptophysin and sybII retrieval within nerve terminals suggests that the two proteins are trafficked through the same pathway. What is of interest is that this correlation between sybII and synaptophysin retrieval was retained with the T198I mutation, even though less sybII was retrieved relative to synaptophysin at individual nerve terminals. This suggests that parallel mechanisms may be present to recover sybII from the plasma membrane even in the potential disruption of its interaction with synaptophysin (Koo et al., 2015, Koo et al., 2011). The interaction between synaptophysin and sybII has been identified for many years (Calakos and Scheller, 1994, Edelmann et al., 1995, Washbourne et al., 1995), however little definitive information is present relating to exactly where the interaction occurs. Previous work involving in vitro and in vivo truncation studies suggests that sybII interacts with synaptophysin via its transmembrane domain and flanking N-terminal sequence (Felkl and Leube, 2008, Reisinger et al., 2004, Yelamanchili et al., 2005). Synaptophysin may also interact with sybII via its transmembrane domains, since removal of its cytoplasmic C-terminus does not impact on sybII binding (Bonanomi et al., 2007, Felkl and Leube, 2008). Recent structural studies have suggested that the 4th transmembrane domain of synaptophysin is critical for the sybII interaction. (Adams et al., 2015). The previously identified G217R mutation may disrupt this interaction since it is predicted to disrupt a transmembrane binding pocket for the transmembrane domain of sybII (Adams et al., 2015). Since T198I mutation is juxtaposed to 4th transmembrane domain in the SV lumen it may have a similar effect on sybII binding. However the divergence in their effect on sybII plasma membrane stranding suggest that a simple loss of sybII interaction cannot be solely responsible for their effects on sybII trafficking.

*SYP* is an X-linked gene making the male patient hemizygous for this mutation, therefore our molecular replacement strategy is representative of the clinical condition. How could such a profound defect in the trafficking of a key cargo molecule such as sybII result in the survival of an individual carrying this mutation, albeit with a severe neurodevelopmental disorder? Firstly there is a large excess of sybII on SVs. There are approximately 70 copies of sybII on an average SV (Takamori et al., 2006, Wilhelm et al., 2014) whereas only between one to three copies are essential for SV fusion (Mohrmann et al., 2010, Sinha et al., 2011, van den Bogaart et al., 2010). One would therefore predict that this activity-dependent defect in sybII retrieval would only impact on neurotransmission during repeated bursts of intense activity. In support, we have observed a decrease in presynaptic performance during repeated patterns of activity in synaptophysin knockout neurons (unpublished observations). Given that the localisation of sybII is unchanged in resting neurons, the T198I mutation may only impact on a specific subset of neurons that fire at sufficient frequencies to render them susceptible to dysfunctional sybII retrieval. In support, defective sybII retrieval was more pronounced in inhibitory neurons from mice where the specific sybII adaptor AP180 was deleted (Koo et al., 2015). Importantly, these mice showed a greater impairment in hippocampal inhibitory neurotransmission, resulting in inhibitory/excitatory imbalance, an outcome that has been linked to several neurodevelopmental disorders (Koo et al., 2015). It is possible therefore that the impaired sybII retrieval observed with this synaptophysin mutation results in a similar defect. Therefore the identification of activity patterns that render key neurons and circuits susceptible to specific presynaptic mutations may be central to understanding the genesis of specific neurodevelopmental disorders.
